# Mycosis Due to Scopulariopsis Koningi

**Published:** 1916-10

**Authors:** 


**Mycosis Due to Scopulariopsis Koningi.** A. Sartory, Paris, Le Prog. Med., July 5. A milkman presented two gummatous lesions of the right thigh. Syphilis and tuberculosis were excluded. Mycelial filaments were found in the odorless, creamy pus and a fungus was cultivated, identified as above. Raymond and Parisot, Contes Hendues, de l'Acad. des Sciences, May, declare that this organism is involved in freezing of the feet.

